# Variation in Performance Strategies of a Hand Mental Rotation Task on Elderly

**DOI:** 10.3389/fnhum.2019.00252

**Published:** 2019-07-19

**Authors:** Izumi Nagashima, Kotaro Takeda, Nobuaki Shimoda, Yusuke Harada, Hideki Mochizuki

**Affiliations:** ^1^Department of Occupational Therapy, Faculty of Health Sciences, Kyorin University, Mitaka, Japan; ^2^Faculty of Rehabilitation, School of Healthcare, Fujita Health University, Toyoake, Japan; ^3^Department of Rehabilitation, Faculty of Health Sciences, Tokyo Kasei University, Sayama, Japan

**Keywords:** mental rotation, old people, motor imagery, visual imagery, reaction time, handedness

## Abstract

A hand mental rotation task (HMRT) is a task wherein a person judges whether an image of a rotated hand is of the right or left hand. Two performance strategies are expected to come into play when performing these tasks: a visual imagery (VI) strategy, in which an image is mentally rotated, and a motor imagery (MI) strategy, in which the movement of a person’s own hand is simulated. Although elderly people generally take some time to perform these tasks, ability differs greatly between individuals. The present study hypothesizes that there is a relationship between differences in task performance strategy and performance ability, and it compares performance strategy among elderly people divided into groups with a short mental rotation time and a long mental rotation time. In response to images of the palm, both groups displayed a medial-lateral effect in which responses were faster for images where the third finger was rotated toward the midline of the body than images rotated in the opposite direction, and we inferred that an MI strategy was primarily employed. Meanwhile, in response to images of the back of the hand, a medial-lateral effect was also observed in the group with a long mental rotation time and not in the group with the shortest mental rotation time (VI strategy). These results suggest that different strategies for performing HMRT task are used by elderly people with a short mental rotation time and those with a long mental rotation time.

## Introduction

A hand mental rotation task (HMRT) is a task in which a person judges whether an image of a rotated hand is showing the right or left hand. Many previous studies have demonstrated a medial-lateral effect (De Simone et al., 2013) in which response time (RT) is shorter when a participant’s hand is in a relatively mobile orientation when actually laid over the presented image of a hand, meaning the image is of a hand rotated in a medial orientation with the fingertips in the image facing the midline of the body, and RT is longer when the image is of a hand rotated in the opposite way, the relatively immobile lateral orientation (Parsons, 1987; Saimpont et al., 2009; Takeda et al., 2010; ter Horst et al., 2010; Bläsing et al., 2013; Zapparoli et al., 2014, 2016; Nagashima et al., 2017). Based on the fact that the dorsal intraparietal sulcus and the premotor cortex are active during the performance of these tasks, de Lange et al. (2006) states that motor imagery (MI) is latently and personally induced.

Studies on aging using HMRT show that RT is longer in elderly people than in younger people. This is thought to be caused by factors like the deterioration of motor simulation abilities based in declining bodily processes (Saimpont et al., 2009) and declining motor planning abilities (De Simone et al., 2013). Furthermore, studies have also shown that elderly people tend to sacrifice performance speed on cognitive tasks to maintain the accuracy of their answers (Endrass et al., 2012; Lamb et al., 2016).

It has been reported that the abilities of elderly people are not necessarily worse than those of younger people, with 25% of elderly people being able to perform a forced choice reaction task in the same amount of time as younger people (Costello and Bloesch, 2017). Task performance strategies for HMRT are not limited to MI. Previous researches suggest that, in HMRT using images of the back of the hand, younger people use a strategy of visually rotating hand images (visual imagery; VI strategy; ter Horst et al., 2010; Bläsing et al., 2013; Zapparoli et al., 2014, 2016) just as in mental rotation tasks using three-dimensional objects (Shepard and Metzler, 1971) or alphanumeric characters (Cooperau and Shepard, 1973) as stimuli, and that elderly people may use MI in combination with VI strategy (Nagashima et al., 2017). Because aging creates more variation in function and ability between individuals (Buczylowska and Petermann, 2018), elderly people may be a population that includes individuals with varying task performance abilities and strategies. This led us to hypothesize that elderly people with short and long mental rotation times in HMRT might have different task performance strategies, and we investigated differences in the presence of the medial-lateral effect in groups divided by mental rotation time.

## Materials and Methods

### Participants

The participants were 106 right-handed elderly people (ages 65–88). We used a self-administered questionnaire to confirm that participants had no past or present central nervous system disorders or mental illnesses, and no current upper limb or visual disorders. The objectives and methods of this study were explained to participants verbally and in writing. All subjects gave written informed consent in accordance with the Declaration of Helsinki. Approval of the protocol was obtained from the ethics committee of the Kyorin University Ethical Review Board (approval number: 27-32).

### Experimental Procedure

**Figure 1:**
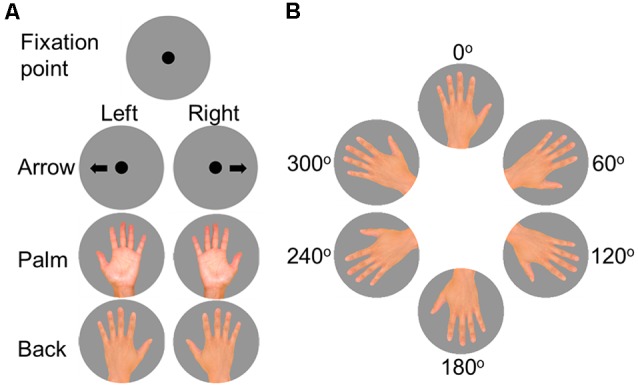
**(A)** A fixation point and arrows directed left and right were used for the left-right choice task. The fixation point and hand images (with all fingers spread) of the palm and back of the hand of both sides were used for the hand mental rotation task (HMRT). **(B)** The rotated angle of the hand image in the present study was set at six orientations in 60° steps.

**Figure 2:**
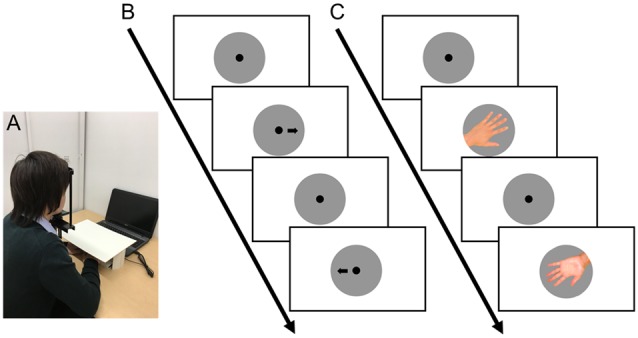
**(A)** Experimental setup. The face direction was mildly kept in position using a chin and head rest. The participants, whose hands were covered by a board in order not to refer to their own hand while performing the task, were required to press the F and J keys for left and right judgment, respectively. In the left-right choice task **(B)** and in the HMRT **(C)**, a fixation point was first displayed for 1.5 s, and then an arrow with fixation point **(B)** or a rotated hand image **(C)** was presented until the participants pressed the key. Time from the image presentation to key pressing was measured as the response time (RT).

In a quiet room, participants first performed a left-right choice task using an image of an arrow pointing left or right. Next, they performed an HMRT to determine whether an image of a hand was of the left or right hand. Figure 1 shows the fixation, arrow, and hand images. Before this HMRT task, they first practiced using six images of hands. The participants were seated in front of a laptop computer with a 15.6-inch screen (Latitude 15 3000 Series, Dell-Japan Corp., Kawasaki, Japan), with the second finger of the left hand on the F key of an external keyboard (TK-FCP026BK, ELECOM Corp., Osaka, Japan) and the second finger of the right hand on the J key. Furthermore, the participants’ hands were hidden from view by a cover (Figure 2A). In this task, a fixation point with a 3-cm diameter was displayed on screen for 1.5 s, after which one stimulus image was presented randomly. Participants were instructed to determine whether an arrow was facing left or right or whether a hand was the left or right hand, pressing the F key if left and the J key if right. They were asked to press the appropriate key as quickly as possible while still being accurate. The image disappeared when the participant pressed a key and, after displaying the fixation point, the next image was presented. The time courses of the two tasks are shown in Figures 2B,C. We recorded the accuracy and RT from the time the stimulus image was displayed until a key was pressed. E-Prime 2.0 (Psychology Software Tools, Inc., Pittsburgh, PA, USA) was used to display the images and record the measurements.

In the left-right choice task using arrow images, left and right arrows were presented 15 times each. To account for participants getting used to the task, the RT was averaged for left and right arrows respectively for trials in which the answer in the 8th to 15th arrow displayed was correct, and this average was used as the motor response generation time for participants.

The HMRT used images of hands with all fingers spread in which the third finger was pointed upward at an angle of 0° and moved clockwise in increments of 60°, resulting in six different positions. A total of 96 images was used: left hand/right hand × back of hand/palm × 6 display angles × 4 repetitions. Mental rotation time (ΔRT) was calculated by subtracting the motor response generation time for arrows with the same orientation from the RT of hand images (Zapparoli et al., 2016; Nagashima et al., 2017). This study analyzed medial images with the third finger facing the midline of the body (images of the left hand at 60° and 120° and the right hand at 240° and 300°) and lateral images with the hand facing the opposite direction (images of the left hand at 240° and 300° and the right hand at 60° and 120°), comparing the ΔRT of presentation orientation (medial and lateral) in images of the back of the hand and the palm.

### Statistical Analyses

For both tasks, only correct responses were used in the statistical analyses. Based on the distribution of average ΔRT values for medial and lateral images of the back of the hand and the palm, participants were divided into four groups using quartiles according to mental rotation time (Table 1, Group A, B, C, and D in order of the fastest to the slowest). The sex ratios of the groups were compared with a chi-squared test. Age, laterality quotient (LQ; Oldfield, 1971), years of education, RT and accuracy for the left-right choice task, and HMRT accuracy in the groups were compared using one-way analysis of variance (ANOVA). The Bonferroni correction was used for each *post hoc* test.

**Table 1:** Participants in each group (mean ± standard deviation).

	Group (*n*)/range and mean of ΔRT for hand (s)				
	A (26) <0.99 0.72 ± 0.16	B (27) 0.99–1.29 1.13 ± 0.10	C (27) 1.29–1.97 1.60 ± 0.20	D (26) 1.97< 2.85 ± 0.70	*p*-value
Male/Female	14/12	11/16	10/17	15/11	0.365
Age (years)	75.4 ± 6.03	78.0 ± 6.26	76.4 ± 6.64	75.8 ± 5.94	0.437
LQ	95.8 ± 8.09	95.2 ± 8.49	98.5 ± 4.56	95.7 ± 9.48	0.403
Education (years)	12.3 ± 2.36	12.6 ± 2.50	13.0 ± 2.28	13.7 ± 2.59	0.206
RT for arrow (s)
Left	0.33 ± 0.07*^‡^	0.40 ± 0.12	0.35 ± 0.05*	0.42 ± 0.09	0.001
Right	0.32 ± 0.06*	0.36 ± 0.08	0.35 ± 0.06*	0.41 ± 0.09	0.000
Accuracy rate for arrow (%)
Left	100.0 ± 0.0	99.1 ± 3.3	99.1 ± 3.3	98.6 ± 4.1	0.413
Right	98.6 ± 4.1	98.6 ± 4.0	99.1 ± 3.3	100.0 ± 0.0	0.369
HMRT accuracy (%)	94.8 ± 4.37^†^	91.0 ± 7.20	89.9 ± 8.29	87.6 ± 8.82	0.007

LQ, laterality quotient. RT, response time. *Significantly shorter than group D. ^†^Significantly higher than group D. ^‡^Significantly shorter than group B.

For each group, two-factor repeated measure ANOVA was performed for the within-subject factors back and palm (back/palm) and presentation orientation (medial/lateral) to compare the difference in ΔRT. SPSS Statistics (Ver. 24.0, IBM Corporation, Armonk, NY, USA) was used. The significant level for ANOVA in each group was 0.0125 (Bonferroni correction for the four groups).

## Results

In terms of demographics within groups, no significant differences were observed in gender ratio, age, LQ, or years of education. In the left-right choice task for arrows, a significant main effect was observed for both left and right arrows (Left, *F*_(3,102)_ = 6.40, *p* = 0.001; Right, *F*_(3,102)_ = 7.42, *p* = 0.000; results of subordinate tests are shown in Table 1). A significant main effect was observed for the accuracy rate in HMRT (*F*_(3,102)_ = 4.305, *p* = 0.007), and the fastest group (Group A) had a significantly higher rate than the slowest group (Group D, *p* = 0.004).

**Figure 3:**
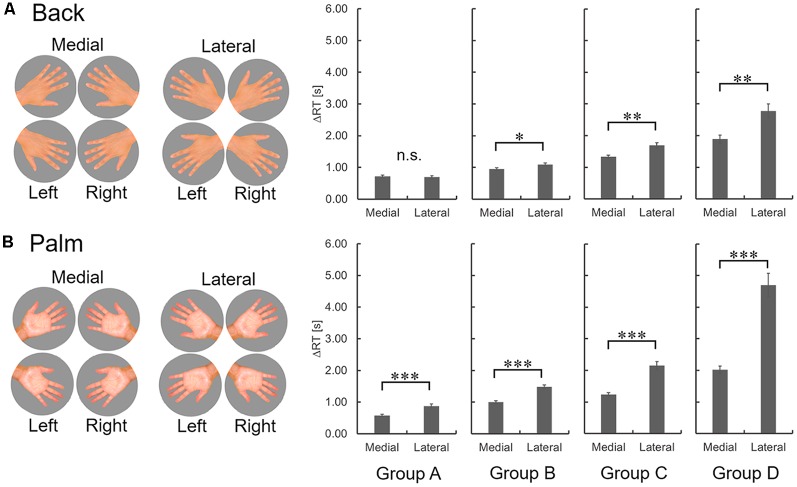
Medial and lateral hand images and difference of ΔRT in each group for the back **(A)** and palm **(B)**. The error bars indicate the standard error of the mean. n.s., not significant. *p < 0.05, **p < 0.01, ***p < 0.001.

A significant interaction was observed between back/palm and medial/lateral in the ΔRT of each group (A, *F*_(1,25)_ = 31.59, *p* = 0.000; B, *F*_(1,26)_ = 16.69, *p* = 0.000; C, *F*_(1,26)_ = 15.04, *p* = 0.001; D, *F*_(1,25)_ = 33.04, *p* = 0.000). In terms of the ΔRT for back-of-the-hand images, no significant difference was observed in Group A, while groups B, C, and D demonstrated a medial-lateral effect in which the response to medial pictures was faster than the response to lateral pictures (Figure 3A). Meanwhile, for palm images, a medial-lateral effect was observed in all groups (Figure 3B).

## Discussion

The results demonstrated that the group of elderly people with a short mental rotation time used different performance strategies for the palm and the back of the hand. The fact that no medial-lateral effect was observed in Group A for mental rotation time of back-of-the-hand images suggests not that the elderly people in these groups did not simulate their own body movements (MI strategy), but that they visually rotated images (VI strategy), just as in mental rotation tasks using three-dimensional objects (Shepard and Metzler, 1971) or alphanumeric characters (Cooperau and Shepard, 1973). This is the same strategy that younger people use to perform HMRT for back-of-the-hand images (ter Horst et al., 2010; Bläsing et al., 2013; Zapparoli et al., 2014). A study on younger people demonstrated that RT for back-of-the-hand images was shorter than RT for palm images and shows led to stronger activity in the left cuneus and precuneus of the occipital lobe (Zapparoli et al., 2014). As such, we speculate that the elderly people in Group A have maintained their ability to process visual information.

Previous studies revealed that young people perform HMRT for back-of-the-hand images predominantly using a VI strategy and for palm images with an MI strategy (ter Horst et al., 2010; Bläsing et al., 2013; Zapparoli et al., 2014). On the other hand, elderly people, including ΔRT with a median level of performance (groups B–D) showed a medial-lateral effect for back-of-the-hand images in the present study. It suggests that most elderly people perform the HMRT involving back-of-the-hand images predominantly using an MI strategy, unlike young people. In elderly people, in general, even if their visual and cognitive functions are within the normal range, their ability to process visual information declines (Mahoney et al., 2012; Guest et al., 2015; Owsley, 2016). The time taken for mental rotation tasks using three-dimensional objects or alphanumeric characters, which are performed using a VI strategy, is also prolonged (Gaylord and Marsh, 1975; Jacewicz and Hartley, 1987). However, in the elderly people of groups B–D, the performance strategy itself was different rather than poorer visual information processing ability. In hand mental rotation studies, RT is longer for the MI strategy than for the VI strategy (ter Horst et al., 2010; Bläsing et al., 2013; Zapparoli et al., 2014). As for the RT profile concerning the back-of-the-hand in groups B to D (Figure 3A), the medial-lateral effect (the difference between ΔRT for medial and lateral) tended to be stronger in the slower performance group. Regarding the RT profile concerning the palm of the hand in the present study, the medial-lateral effect and its increasing tendency could also be seen in all the elderly groups (Figure 3B). Because motor simulation abilities are correlated with actual motion (Decety et al., 1989), the actual motor functions may be reflected in the grouping of the present study. It might be necessary to investigate the actual upper limb motor function of participants in the present study. In addition, although motor function declines and interindividual differences increase with age (Hunter et al., 2016), atrophies of the primary sensorimotor areas are less than those of memory- and imagination-related brain areas (Fjell et al., 2013). Therefore, many elderly people may have implicitly chosen the MI strategy over VI strategy even in the HMRT of the back-of-the-hand.

MI may contribute to improving motor impairments after stroke because it activates the precentral gyrus and supplementary motor area (Jeannerod and Decety, 1995; Hétu et al., 2013). However, it is difficult to control and identify the imagery of the patients explicitly. Since the HMRT has the potential to elicit the participant’s own hand movement implicitly, it has been applied in rehabilitation treatment (Moseley, 2004; Polli et al., 2017; Dilek et al., 2018). In order to do this, it must be guaranteed that the MI is induced during HMRT. Actually, in the present study, elderly people did not always perform HMRT using an MI strategy, especially for the back-of-the-hand images. Because the MI strategy was predominantly used for the palm of the hand images in all groups, clinical intervention using the palm may be more effective for motor functional improvement. On the other hand, the performance strategy of HMRT might be transformed by the number of rotation axes within the hand image set (more rotational axes facilitate MI engagement; ter Horst et al., 2010) and might differ depending on handedness (Takeda et al., 2010). For the clinical application of HMRT, therefore, a more extensive collection of evidence in the hand-image presentation paradigm and in the characteristics of the patients or elderly people is necessary. In addition, behavioral analysis alone, including the present study, may be limited in identifying performance strategy. The combination of neurophysiological basis such as quantification of event-related desynchronization involved in motor image process (Chen et al., 2013) seems to be more important.

Many studies of HMRT have focused on the relationship between the rotated angle of the hand images and RT (RT profile) depending on the palm/back of the hand and/or direction of the fingertip (Parsons, 1987; Saimpont et al., 2009; Takeda et al., 2010; ter Horst et al., 2010; Bläsing et al., 2013). The RT profile for the HMRT in these studies includes visual encoding, mental rotation, comparison, decision making, and motor response generation (Seurinck et al., 2004). In the present study, we have subtracted the motor response generation time (by the left/right judgment for arrow image) from the RT for the HMRT. This subtraction was also performed in a previous study by Zapparoli et al. (2016). The motor response generation time is significantly prolonged by aging (Yordanova et al., 2004; Kolev et al., 2006). Interindividual differences are also increased with age (Fozard et al., 1994; Der and Deary, 2006). Actually, the participants in group A performed the left-right choice task with a shorter RT than others. Therefore, we argue that the subtraction of the motor generation process is important especially for the study of elderly people to assess the cognitive process of the hand mental rotation.

The fact that Group A had the shortest RT with high accuracy rates in both arrow and hand tasks does not mean that the elderly people in Group A sacrificed accuracy to increase response speed. When it comes to the performance of visual rotation, studies show a strong relationship with stimulation of the prefrontal cortex (Carpenter et al., 1999) and important involvement from central executive networks including the inferior frontal gyrus and middle frontal gyrus on both sides (Gao et al., 2017). As such, there is a strong possibility that Group A included many high-performing elderly people (Fjell et al., 2006) who had maintained the function of their frontal cortex. Future research regarding the relationship between HMRT performance ability and cognitive function tests may be needed.

## Data Availability

The datasets generated for this study are available on request to the corresponding author.

## Ethics Statement

All subjects gave written informed consent in accordance with the Declaration of Helsinki. Approval of the protocol was obtained from the ethics committee of the Kyorin University Ethical Review Board (approval number: 27-32).

## Author Contributions

IN, KT, NS, YH, and HM conceived the presented idea and designed the experiments. IN and YH carried out the experiments. IN, YH, and HM analyzed the data. IN, KT, NS, and HM interpreted the results and drafted the manuscript.

## Conflict of Interest Statement

The authors declare that the research was conducted in the absence of any commercial or financial relationships that could be construed as a potential conflict of interest.
